# Evolutionary and functional analysis of the DIR gene family in Moso bamboo: insights into rapid shoot growth and stress responses

**DOI:** 10.3389/fpls.2025.1535733

**Published:** 2025-02-25

**Authors:** Xueyun Xuan, Shiying Su, Jialu Chen, Jiaqi Tan, Zhen Yu, Yang Jiao, Sijia Cai, Zhijun Zhang, Muthusamy Ramakrishnan

**Affiliations:** ^1^ State Key Laboratory of Subtropical Silviculture, Bamboo Industry Institute, Zhejiang A&F University, Hangzhou, Zhejiang, China; ^2^ State Key Laboratory of Tree Genetics and Breeding, Co-Innovation Center for Sustainable Forestry in Southern China, Bamboo Research Institute, Key Laboratory of National Forestry and Grassland Administration on Subtropical Forest Biodiversity Conservation, School of Life Sciences, Nanjing Forestry University, Nanjing, Jiangsu, China

**Keywords:** DIR gene family, rapid shoot growth, hormone response, abiotic stress, TFs regulatory network, Moso bamboo

## Abstract

Dirigent (DIR) proteins are key regulators of lignin and lignan biosynthesis and play critical roles in plant hormone responses, abiotic stress tolerance, and growth and development. This study identified and characterized 47 *PeDIR* genes in Moso bamboo, classifying them into three groups. Phylogenetic and comparative analyses revealed strong evolutionary conservation, with the Moso bamboo *PeDIR* genes being most closely related to those in rice and maize. DIR proteins within each subfamily exhibited high conservation in motif composition, domain structure, and 3D configuration. Subcellular localization and protein interaction studies further elucidated *PeDIR* gene functions. Specifically, PeDIR02 primarily localized to the cell membrane and was shown to be unable to form homodimers in yeast two-hybrid (Y2H) assays. Transcriptome and expression analyses revealed the involvement of *PeDIR* genes in rapid shoot growth, indicating roles in lignin biosynthesis and cell wall modification. Transcriptome and qRT-PCR data also demonstrated the responsiveness of these genes to hormones and abiotic stresses, such as drought and salinity. This study constructed the first comprehensive regulatory network between transcription factors (TFs) and *PeDIR* genes, identifying ERF, DOF, and MYB TFs as key synergistic regulators of *PeDIR* gene expression.

## 1 Introduction

Moso bamboo (*Phyllostachys edulis*), the most extensively cultivated species in the Bambusoideae subfamily, holds substantial ecological and economic significance in China and Asia (Peng et al., 2013; Li et al., 2015; Yu et al., 2019; Huang et al., 2022). However, its rapid growth and development are often constrained by environmental factors such as salinity, drought, and low temperatures. These factors not only reduce timber production and shoot yield but also threaten the overall stability of Moso bamboo forests (Liu et al., 2019; Ramakrishnan et al., 2020). Understanding the genetic mechanisms underlying stress resistance is therefore crucial for developing more resilient bamboo varieties.

Dirigent (DIR) proteins, first discovered in weeping forsythia (*Forsythia suspense*), play key roles in lignin and lignan biosynthesis (Davin et al., 1997; Gang et al., 1999; Burlat et al., 2001). Through their highly conserved dirigent domain, DIR proteins precisely capture and orient E-coumaryl alcohol radical intermediates, enabling stereoselective coupling of lignin and lignan precursor compounds in the presence of auxiliary oxidative enzymes (Gang et al., 1999; Burlat et al., 2001). During lignin and lignan biosynthesis, they mediate site-specific radical coupling reactions, guiding the formation of specific stereoisomers, such as (+)-pinoresinol and (−)-pinoresinol, through a spatial orientation template (Kim et al., 2002; Dalisay et al., 2015). Lignans, a class of secondary metabolites widely distributed in plants, exhibit notable antifungal activity by inhibiting pathogen growth and spread, thereby enhancing plant resistance to external stresses (Harmatha and Dinan, 2003; Zhang et al., 2022a). Lignin, a major component of plant cell walls, provides structural support, participates in plant defense mechanisms, regulates growth and development, and exhibits antifungal activity, further enhancing plant resistance to external stresses. The *DIR* gene family has been characterized in various plants, including *Arabidopsis thaliana* (Paniagua et al., 2017), *Oryza sativa* (rice) (Liao et al., 2017), *Solanum tuberosum* (Jia et al., 2023), *Picea* spp (Ralph et al., 2006), and Gossypium spp (Zhengwen et al., 2021), and is categorized into six subfamilies: DIR-a, DIR-b/d, DIR-c, DIR-e, DIR-g, and DIR-f (Luo et al., 2022). Among these, the DIR-a subfamily comprises proteins responsible for stereoselective lignin and lignan formation, referred to as dirigent genes. The functions of other subfamilies remain unclear and are termed *DIR*-like genes (Ralph et al., 2006, 2007).

DIR proteins are found in many vascular plants and play critical roles in growth, development, and resistance to environmental stresses and diseases (Jin-long et al., 2012; Thamil Arasan et al., 2013). Their defining feature is the highly conserved dirigent domain, which facilitates substrate binding and is essential for specific biochemical reactions (Pickel et al., 2012). Most DIR proteins have a dirigent domain with a three-dimensional structure composed of β-sheets (Li et al., 2024a). Some DIR proteins possess two tandem dirigent domains, while others have an additional jacalin domain (Kim et al., 2015; Gong et al., 2023; Jiang et al., 2024). At the genomic level, DIR genes exhibit structural diversity. Most *DIR* genes lack introns, but a subset contains one or two introns (Dong et al., 2021; Zhang et al., 2022a).

Research indicates that DIR proteins have diverse roles in plants, including tolerance to abiotic stresses and responses to environmental challenges (Chen et al., 2024). Notably, *DIR* genes often show significant responses to stress-related hormone induction and abiotic stress conditions. For example, in sugarcane, *ScDIR11* and *SHDIR16* expression are significantly upregulated by salicylic acid (SA) and jasmonic acid (JA) treatments and exhibit transcriptional increases under salinity, drought, and hydrogen peroxide (H_2_O_2_) stress (Jin-long et al., 2012). In soybeans, *GmDIR22* expression is significantly upregulated in response to gibberellins (GA), SA, methyl jasmonate (MeJA), and abscisic acid (ABA) (Li et al., 2017).

Similarly, in peppers, *CaDIR2*, *CaDIR3*, *CaDIR6*, *CaDIR7*, and *CaDIR11* are upregulated in response to MeJA, SA, NaCl, and mannitol treatments. Notably, the absence of *CaDIR7* reduces root vitality, defense capabilities, and resistance to salinity and diseases (Khan et al., 2018). In eggplants, several *DIR* genes, such as *SmDIR3*, *SmDIR6*, *SmDIR12*, and *SmDIR22*, are downregulated under high temperatures, with varying expression levels at different stages. *SmDIR5* and *SmDIR22* are repressed under low temperatures, and *SmDIR22* expression decreases under salt stress (Zhang et al., 2022a). Furthermore, *DIR* genes in pear (Cheng et al., 2018), alfalfa (Song and Peng, 2019), and tobacco (Li et al., 2024b) strongly respond to diverse hormones and abiotic stresses. *DIR* genes also contribute to plant growth and development. For example, the *ESB1* protein with a dirigent domain aids in Casparian strip formation and maintains root barrier integrity (Hosmani et al., 2013).

Understanding the roles of *PeDIR* genes is crucial for revealing Moso bamboo’s defense mechanisms against abiotic stresses and hormone signaling (Saddique et al., 2024). This study systematically analyzed *PeDIR* gene structures, phylogenetic relationships, promoter elements, and collinearity across the genome using bioinformatics tools. A regulatory network linking *PeDIR* genes with upstream transcription factors (TFs) was established. This study also explored *PeDIR* gene expression under hormone treatments, abiotic stresses, and rapid growth, providing insights into their roles in hormone and stress responses in Moso bamboo. These findings lay a foundation for deeper investigation into the functional roles of *PeDIR* genes in stress responses.

## 2 Materials and methods

### 2.1 Identification and physicochemical properties of *PeDIR* genes

Moso bamboo genome data were obtained from the National Center for Gene Research (http://server.ncgr.ac.cn/bamboo/index.php). The DIR protein domain HMM profile (PF03018) was downloaded from the Pfam database (http://pfam.xfam.org/) and used to search the bamboo genome with an e-value threshold of ≤1e^-10^ (Finn et al., 2011). Protein domains were confirmed using the NCBI-CDD database, leading to the identification of PeDIR family members (Letunic et al., 2011; Finn et al., 2015). The molecular weight, theoretical isoelectric point, and hydrophobicity of PeDIR proteins were analyzed using the Expasy ProtParam tool (https://web.expasy.org/protparam/) (Wilkins et al., 1999).

### 2.2 Phylogenetic and synteny analysis of *PeDIR* genes

DIR protein sequences from Moso bamboo, rice, *Zea mays* (maize), and *Brachypodium distachyon* were identified using HMMER3 to search their respective local protein databases. Phylogenetic trees were constructed for both intra- and interspecies analyses using MEGA 11.0. The maximum likelihood method was employed, with 1,000 bootstrap trials to assess tree reliability (Tamura et al., 2021). TBtools was used to align Moso bamboo protein sequences with those from rice, maize, and *B. distachyon*. Chromosomal location data from the four species were combined, and MCScanX was used to identify both intraspecies and interspecies syntenic relationships within the PeDIR gene family. The results were visualized using Circos (Wang et al., 2012).

### 2.3 Gene structure and motif analysis of *PeDIR* genes

The conserved sequence patterns of PeDIR proteins were analyzed using the MEME suite (http://meme-suite.org/) (Bailey et al., 2015). The intron-exon structures of *PeDIR* genes were extracted from the GFF annotation file of the Moso bamboo genome. Using TBtools, the evolutionary relationships, motif patterns, and gene structures of *PeDIR* genes were integrated and visualized.

### 2.4 *Cis*−regulatory elements and construction of *PeDIR* gene regulatory networks

*Cis*-acting elements within the 1.5 kb upstream promoter regions of *PeDIR* genes were identified using PlantCARE (http://bioinformatics.psb.ugent.be/webtools/plantcare/html/) (Lescot et al., 2002). The types and distributions of regulatory elements were visualized with TBtools. Potential transcription factor (TF) binding sites (TFBS) in the 1.5 kb upstream regions of *PeDIR* genes were predicted via PlantPAN (http://plantpan.itps.ncku.edu.tw/plantpan4/index.html) (Chow et al., 2024). TFBS with correlation coefficients above 0.95 were selected for further study. A gene regulatory network based on the identified TFBS was constructed using Cytoscape, and a dynamic network heatmap depicting interactions between *PeDIR* genes and upstream TFs was created using the Omicshare platform (http://www.omicshare.com).

### 2.5 Secondary and tertiary structure prediction of PeDIR proteins

Conserved domains in PeDIR proteins were identified using the CDD-search website (https://www.ncbi.nlm.nih.gov/cdd) and visualized using IBS 2.0 (Xie et al., 2022). Homology modeling of PeDIR proteins was conducted via SWISS-MODEL (https://swissmodel.expasy.org/) (Waterhouse et al., 2018). The 3D structures were refined and optimized using Discovery Studio for improved visualization.

### 2.6 Expression and time-series analysis of *PeDIR* genes based on RNA-seq data

Transcriptome data for Moso bamboo under various treatments were obtained from NCBI to examine *PeDIR* gene expression. The treatments included: (i) seedling roots treated with gibberellin (GA) and naphthalene acetic acid (NAA) (PRJNA413166), (ii) roots treated with abscisic acid (ABA) and salicylic acid (SA) (PRJNA715101), and (iii) roots under drought and high-salinity stress (PRJNA413166). Transcript per million (TPM) values were log2-transformed, and heatmaps of *PeDIR* gene expression levels were created using TBtools (Zhijun et al., 2022; Guo et al., 2024). Additionally, temporal expression patterns of *PeDIR* genes across different shoot heights of Moso bamboo were analyzed using several publicly available transcriptome datasets (PRJNA414226). Short Time-series Expression Miner (STEM) was employed to identify expression trends during the rapid growth stages of bamboo shoots. A trend significance threshold of *p*-value < 0.05 was applied (Ernst and Bar-Joseph, 2006).

### 2.7 Plant materials and experimental treatments

Moso bamboo seeds were sourced from Guilin, Guangxi, China. The seeds were grown in a controlled chamber maintained at 25°C and 70% relative humidity for one month. For hormone treatments, seedling leaves were sprayed with 100 μM ABA or SA solutions and sampled at 0, 3, 6, 12, 24, and 48 hours. Drought stress was induced by exposing the roots to 30% PEG for 3 or 6 hours, while salinity stress was applied by treating the roots with 200 mM NaCl for the same durations. Root samples were collected after each treatment. Untreated samples at 0 hours served as controls. Three biological replicates were collected randomly (Huang et al., 2021).

### 2.8 RNA extraction, reverse transcription, and quantitative reverse-transcription PCR

Total RNA was extracted using the FastPure Plant Total RNA Extraction Kit (Vazyme, China). cDNA synthesis was performed using the HiScript^®^ III 1st Strand cDNA Synthesis Kit. Primers were designed using Beacon Designer 7.0. qRT-PCR was performed on a CFX-96 Real-Time System with three technical replicates per sample. Threshold cycle (CT) values were calculated using the 2^-ΔΔCT method and are presented as mean ± standard deviation (SD). Statistical significance was determined by one-way ANOVA, and data visualization was performed with GraphPad Prism 7 (Livak and Schmittgen, 2001).

### 2.9 Subcellular localization experiments

Subcellular localization predictions for PeDIR proteins were performed using WoLF PSORT (https://wolfpsort.hgc.jp/) (Horton et al., 2007). To confirm this prediction, the full-length CDS of *PeDIR02* was amplified using seamless PCR. The fragments were inserted into the pCAMBIA1300-35S-GFP vector containing the GFP (green fluorescent protein) reporter gene under the 35S promoter. Recombinant plasmids were transformed into *Agrobacterium tumefaciens* strain GV3101 and infiltrated into the abaxial leaves of 5-week-old *Nicotiana benthamiana* plants. The GFP signals were analyzed with a confocal microscope to validate the predicted subcellular localization (Zhang et al., 2022b).

### 2.10 Yeast two-hybrid assay of PeDIR proteins

The CDS of *PeDIR02* was amplified via PCR and cloned into the pGBKT7 and pGADT7 vectors. Yeast two-hybrid (Y2H) experiments were performed using the Matchmaker GAL4 system. Recombinant plasmids were introduced into the AH109 yeast strain and confirmed on SD/-Leu/-Trp medium. Protein interactions were assessed by growing yeast on SD/-Ade/-His/-Trp/-Leu/X-α-Gal medium at 28°C for 3 days. Positive interactions were indicated by colony growth and blue coloration from X-α-Gal breakdown (Ma et al., 2021). The primers used in this study are listed in **Supplementary Tables S1** and **S2**.

## 3 Results

### 3.1 Identification, characterization, and phylogenetic analysis of *PeDIR* genes

A total of 47 *PeDIR* members were identified from the Moso bamboo genome database and were sequentially named *PeDIR01* to *PeDIR47* based on their scaffold arrangements (**Table 1**). The amino acid lengths of these proteins range from 88 for PeDIR47 to 360 for PeDIR14. Their molecular weights vary from 9,934.39 Da for PeDIR47 to 37,524.82 Da for PeDIR14. The predicted isoelectric points range from 4.58 for PeDIR05 to 10.65 for PeDIR16. Hydrophilicity analysis revealed that 17 PeDIR proteins are hydrophilic, while the remaining members are hydrophobic. Subcellular localization predictions indicate that PeDIR proteins are distributed across various subcellular structures, with the majority localized to the cell membrane (31), followed by the chloroplast (7) and the cell wall (5).

**Table 1:** Gene information of *DIR* members in Moso bamboo.

Gene ID	Gene name	Scaffold	Amino acids	PI	Mw (Da)	GRAVY	Prediction Location
PH02Gene38476.t1	*PeDIR01*	Sca2	98	9.65	10914.73	0.245	C
PH02Gene10505.t1	*PeDIR02*	Sca2	198	9.48	21111.09	0.011	Cm
PH02Gene10498.t1	*PeDIR03*	Sca2	103	6.05	11133.76	0.005	Cm
PH02Gene39117.t1	*PeDIR04*	Sca4	113	9.24	13084.03	-0.174	Cw
PH02Gene21871.t1	*PeDIR05*	Sca4	175	4.58	18059.55	0.426	Cm
PH02Gene21872.t1	*PeDIR06*	Sca4	244	5.67	26170.6	0.120	C
PH02Gene41384.t1	*PeDIR07*	Sca4	244	8.76	26058.16	0.121	C
PH02Gene41385.t1	*PeDIR08*	Sca4	228	8.95	23997.6	0.115	C
PH02Gene47479.t1	*PeDIR09*	Sca4	300	7.04	31572.07	0.262	Cm
PH02Gene17742.t1	*PeDIR10*	Sca5	206	5.93	21802.75	0.164	Cm
PH02Gene46616.t1	*PeDIR11*	Sca7	172	5.6	17953.65	0.490	Cm
PH02Gene23777.t1	*PeDIR12*	Sca7	308	9.22	33711.39	-0.167	Cy
PH02Gene23783.t1	*PeDIR13*	Sca7	206	6.94	22740.15	-0.039	C
PH02Gene10604.t1	*PeDIR14*	Sca10	360	5.52	37524.82	0.219	Cm
PH02Gene45653.t1	*PeDIR15*	Sca10	176	4.89	18924.78	0.311	Cm
PH02Gene37326.t1	*PeDIR16*	Sca10	135	10.65	14073.85	-0.191	N
PH02Gene37327.t1	*PeDIR17*	Sca10	205	6.43	21807.9	0.061	Cm
PH02Gene15809.t1	*PeDIR18*	Sca11	199	9.64	21303.26	-0.030	Cm
PH02Gene15808.t1	*PeDIR19*	Sca11	158	9.47	17097.25	-0.177	Cw
PH02Gene03445.t1	*PeDIR20*	Sca12	306	5.9	32972.06	-0.132	Cy
PH02Gene49830.t1	*PeDIR21*	Sca12	179	9.04	19047.7	0.087	Cw
PH02Gene23505.t1	*PeDIR22*	Sca13	198	8.64	20716.51	0.196	Cm
PH02Gene23504.t2	*PeDIR23*	Sca13	187	6.07	19858.14	-0.283	Cm
PH02Gene27724.t1	*PeDIR24*	Sca13	205	5.35	22137.9	-0.039	Cm
PH02Gene27725.t1	*PeDIR25*	Sca13	199	5.92	20953.66	0.132	Cm
PH02Gene49479.t1	*PeDIR26*	Sca13	237	5.64	24866	-0.006	Cm
PH02Gene30332.t1	*PeDIR27*	Sca13	178	6.49	18337.89	0.274	Cm
PH02Gene30333.t1	*PeDIR28*	Sca13	230	7.06	24156.26	-0.069	Cm
PH02Gene30336.t1	*PeDIR29*	Sca13	216	6.59	22872.8	-0.062	Cm
PH02Gene09487.t1	*PeDIR30*	Sca13	153	6.02	16285.49	0.195 .195	Cm
PH02Gene44352.t1	*PeDIR31*	Sca14	333	4.72	32889.13	0.413	Cm
PH02Gene44353.t1	*PeDIR32*	Sca14	340	5.3	34924.64	0.223	Cm
PH02Gene21283.t1	*PeDIR33*	Sca15	180	6.64	18430.08	0.202	Cm
PH02Gene00136.t1	*PeDIR34*	Sca15	345	4.78	34290.51	0.198	Cm
PH02Gene03637.t1	*PeDIR35*	Sca15	231	6.16	23123.46	0.330	Cm
PH02Gene49728.t1	*PeDIR36*	Sca15	155	6.96	16384.66	0.048	C
PH02Gene15063.t1	*PeDIR37*	Sca16	245	9.46	26088.43	-0.137	N
PH02Gene15600.t1	*PeDIR38*	Sca16	318	4.82	31565.48	0.293	Cm
PH02Gene31450.t1	*PeDIR39*	Sca21	188	8.75	19913.88	-0.009	C
PH02Gene26302.t1	*PeDIR40*	Sca21	137	6.25	14481.23	-0.253	Cm
PH02Gene18583.t1	*PeDIR41*	Sca21	348	4.69	34556.86	0.185	Cm
PH02Gene11037.t1	*PeDIR42*	Sca21	280	5.4	28003.89	0.275	Cm
PH02Gene07832.t1	*PeDIR43*	Sca22	305	7	32408.57	-0.015	Cw
PH02Gene20575.t1	*PeDIR44*	Sca23	169	5.52	17835.97	0.192	Cm
PH02Gene44204.t1	*PeDIR45*	Sca24	130	8.68	13952.93	-0.073	Cw
PH02Gene36783.t1	*PeDIR46*	Sca24	184	5.36	19391.13	0.355	Cm
PH02Gene32510.t1	*PeDIR47*	Sca398	88	4.87	9934.39	0.161	Cm

Mw, molecular weight; PI, isoelectric point; GRAVY, grand average of hydropathicity score; Cm, Cell membrane; C, Chloroplast; Cw, Cell wall; N, Nucleus; Cy, Cytoplasm.

### 3.2 Phylogenetic relationships and collinearity analysis of *PeDIR* genes

**Figure 1:**
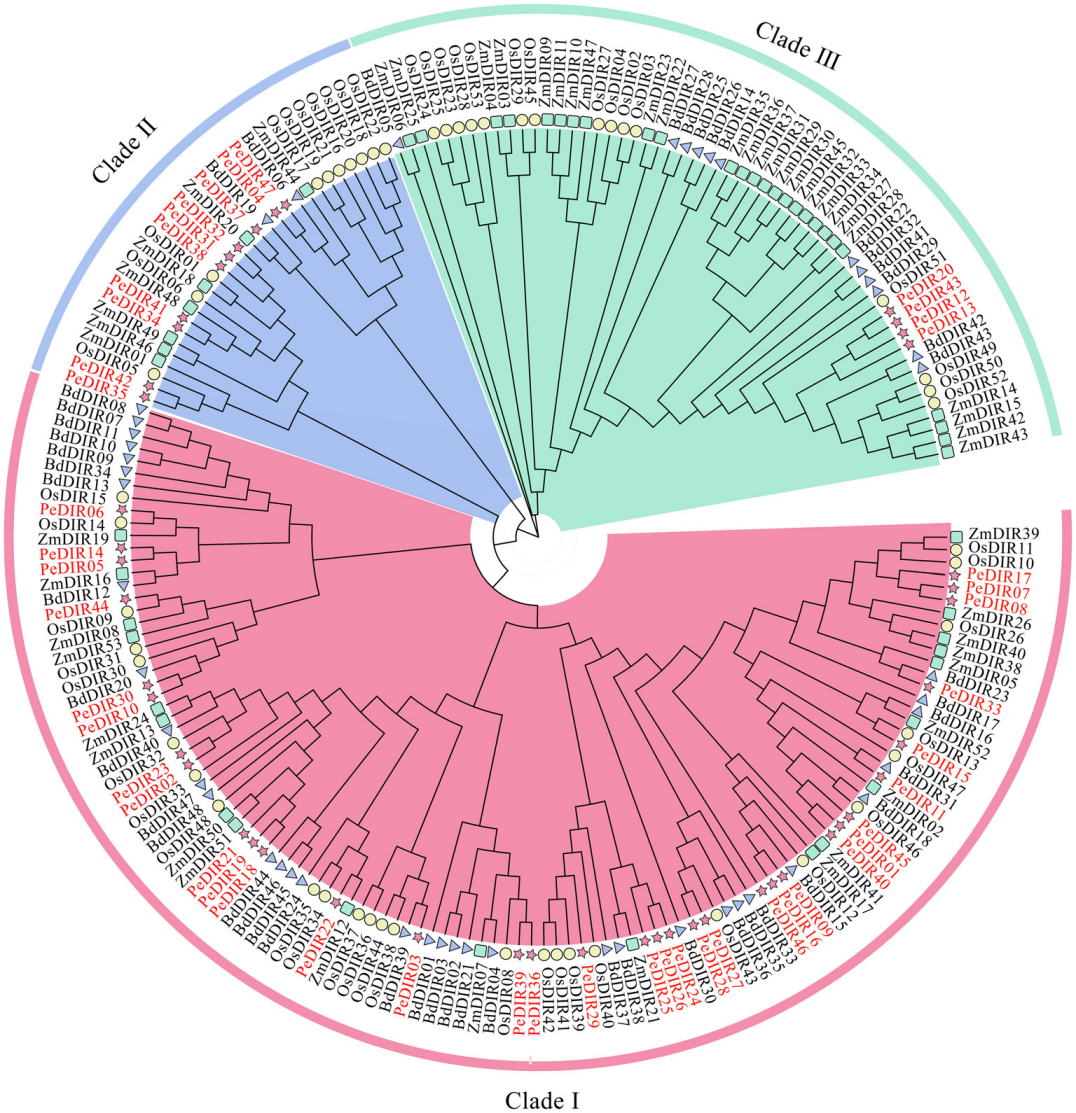
Phylogenetic tree of DIR proteins from *Oryza sativa* (Os, rice), *Zea mays* (Zm, maize), *Brachypodium distachyon* (Bd), and (*Phyllostachys edulis*) (Pe, Moso bamboo).

To explore the evolutionary relationships within the DIR gene family among Poaceae species, rice, maize, and *B. distachyon* were selected as representative species. An interspecies phylogenetic tree was constructed using clustering data from 53 OsDIR, 53 ZmDIR, 48 BdDIR (**Supplementary Table S3**), and 47 PeDIR proteins (**Table 1**). The analysis grouped the *DIR* genes into three distinct classes. The first class contained the most PeDIR proteins (22), followed by the second class with 10, and the third class with only 4 (**Figure 1**). The distribution of PeDIR proteins across subfamilies showed high similarity to DIR members from other Poaceae species, indicating strong conservation of *DIR* genes during Poaceae evolution.

**Figure 2:**
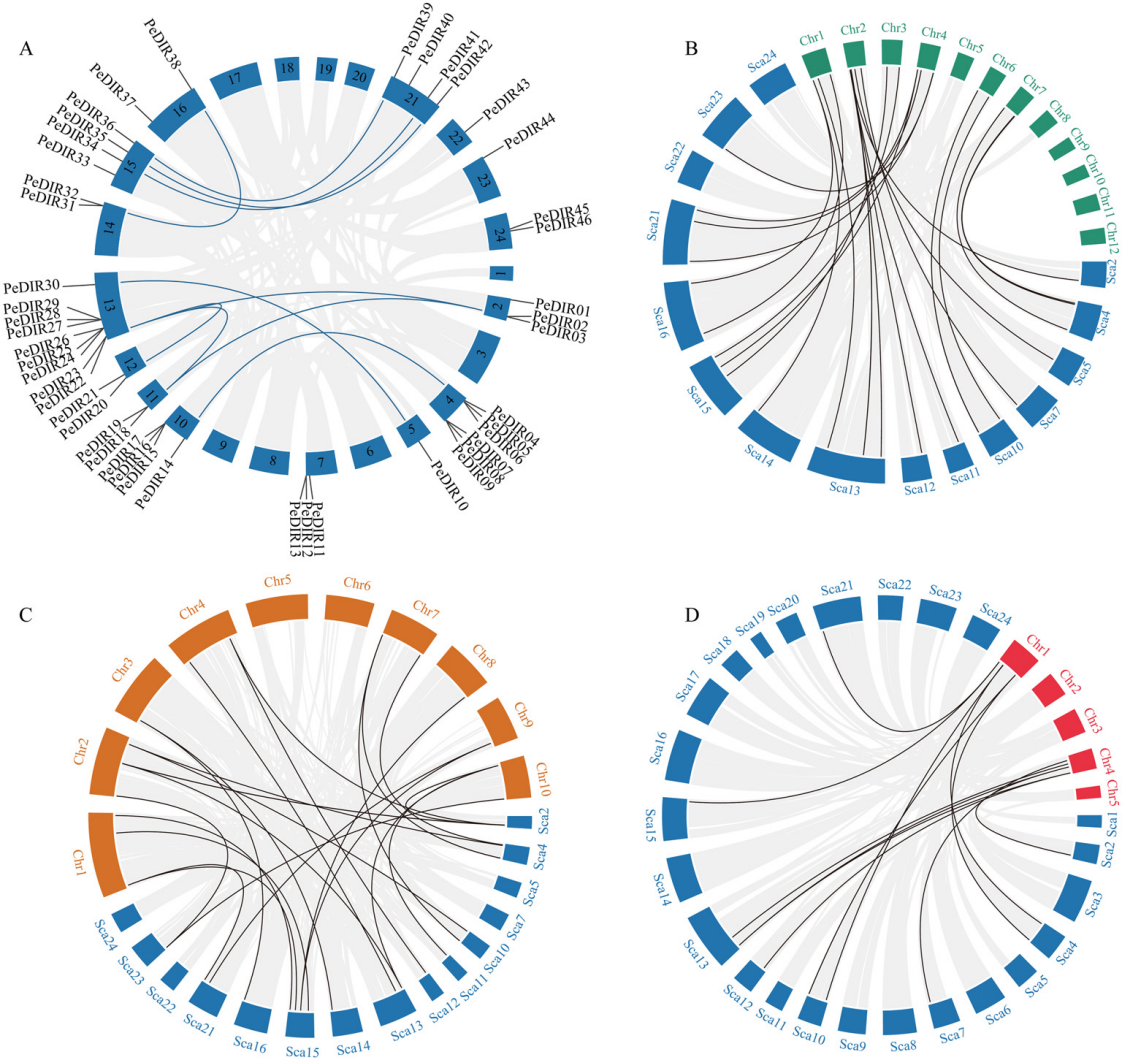
Synteny analysis of *PeDIR* genes. **(A)** Chromosomal distribution and interchromosomal relationships among *PeDIR* genes. **(B–D)** Interspecies collinearity analysis of *DIR genes* (blue for Moso bamboo, green for rice, orange for maize, and red for *Brachypodium distachyon*).

To further investigate the evolutionary dynamics of *PeDIR* genes, intra- and interspecies synteny analyses were performed. According to the Moso bamboo genome annotation, the 47 *PeDIR* genes are distributed across 16 scaffolds, with scaffold 13 containing the highest number of *PeDIR* genes (9). Intraspecies synteny analysis revealed 10 pairs of duplicated *PeDIR* genes (**Figure 2A**). Orthologous gene analysis with other Poaceae species identified 22 gene pairs shared between Moso bamboo and rice, 24 pairs with maize, and 12 pairs with *B. distachyon* (**Figures 2B–D**). These findings suggest that Moso bamboo shares closer evolutionary and phylogenetic relationships with maize and rice, while its relationship with *B. distachyon* is comparatively more distant.

### 3.3 Gene structure and motifs of *PeDIR* Genes

**Figure 3:**
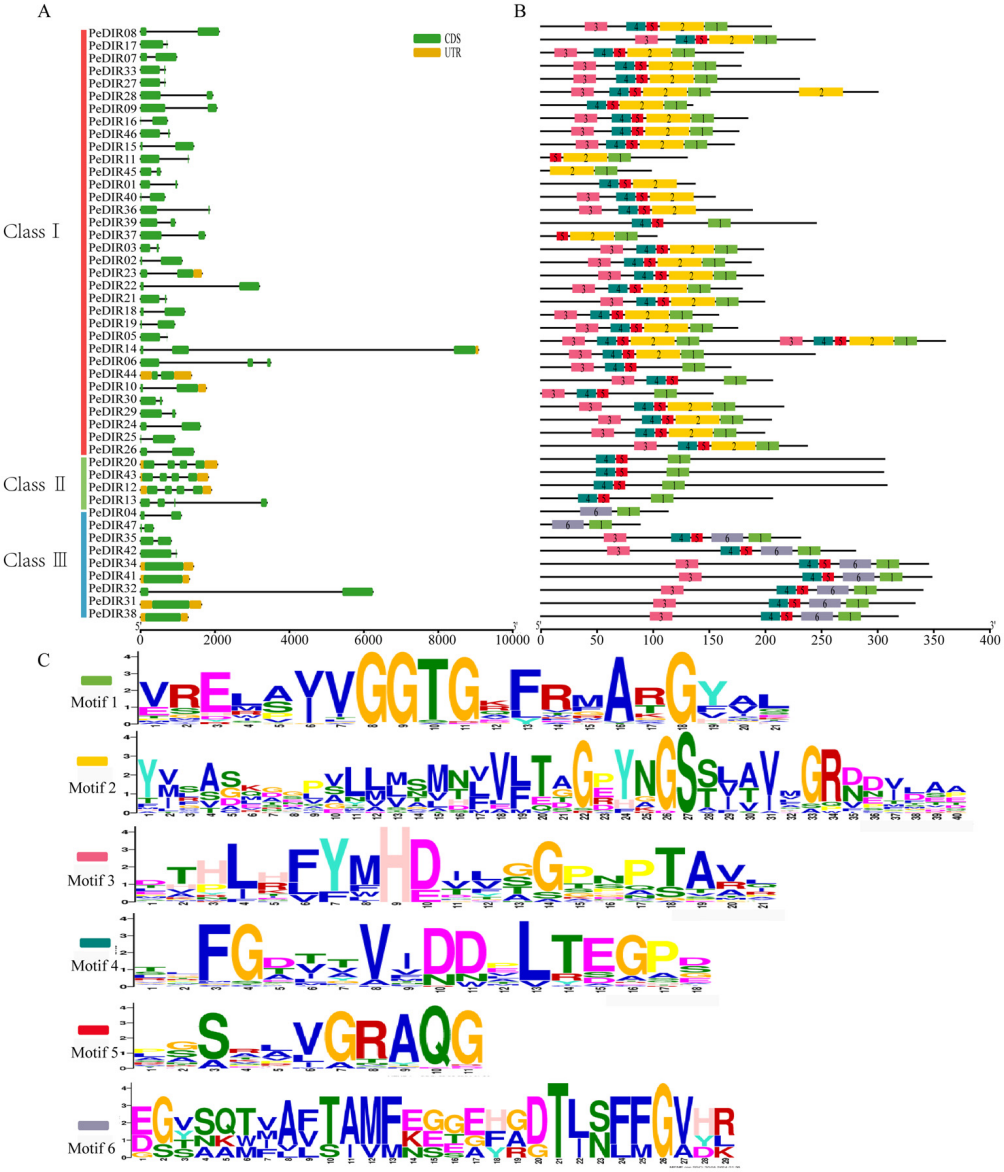
Gene structure and conserved motifs of *PeDIR* genes. **(A)** Gene structure of *PeDIR* genes, showing introns (black lines), exons (yellow rectangles), and untranslated regions (UTRs, green rectangles). **(B)** Conserved motifs of *PeDIR* genes, with motifs 1–6 represented by colored boxes. **(C)** Sequence logos of motifs 1–6.

Intraspecies clustering analysis of PeDIR proteins revealed that Moso bamboo’s *DIR* genes can be grouped into three subfamilies (I, II, III), each exhibiting significant differences in gene structure. Most *PeDIR* genes contain 2–4 exons, except for *PeDIR31, PeDIR34, PeDIR38*, and *PeDIR41* in subfamily III, which are intronless. The remaining *PeDIR* genes have 1–3 introns. Additionally, most *PeDIR* genes, particularly those in subfamily I, lack 5’ and 3’ untranslated regions (UTRs), whereas members of subfamilies II and III generally retain complete UTR structures (**Figure 3A**). Motif analysis revealed that subfamily I members commonly contain motifs 1–5, while subfamily II members are characterized by motifs 1, 4, and 5 only (**Figures 3B, C**). Notably, motif 6 is uniquely present in subfamily III. These findings underscore significant differences in motif distribution and number among the subfamilies. Such differences are consistent with their gene structural characteristics, suggesting that *PeDIR* genes have undergone structural and functional diversification during evolution.

### 3.4 Promoter analysis of *PeDIR* genes

**Figure 4:**
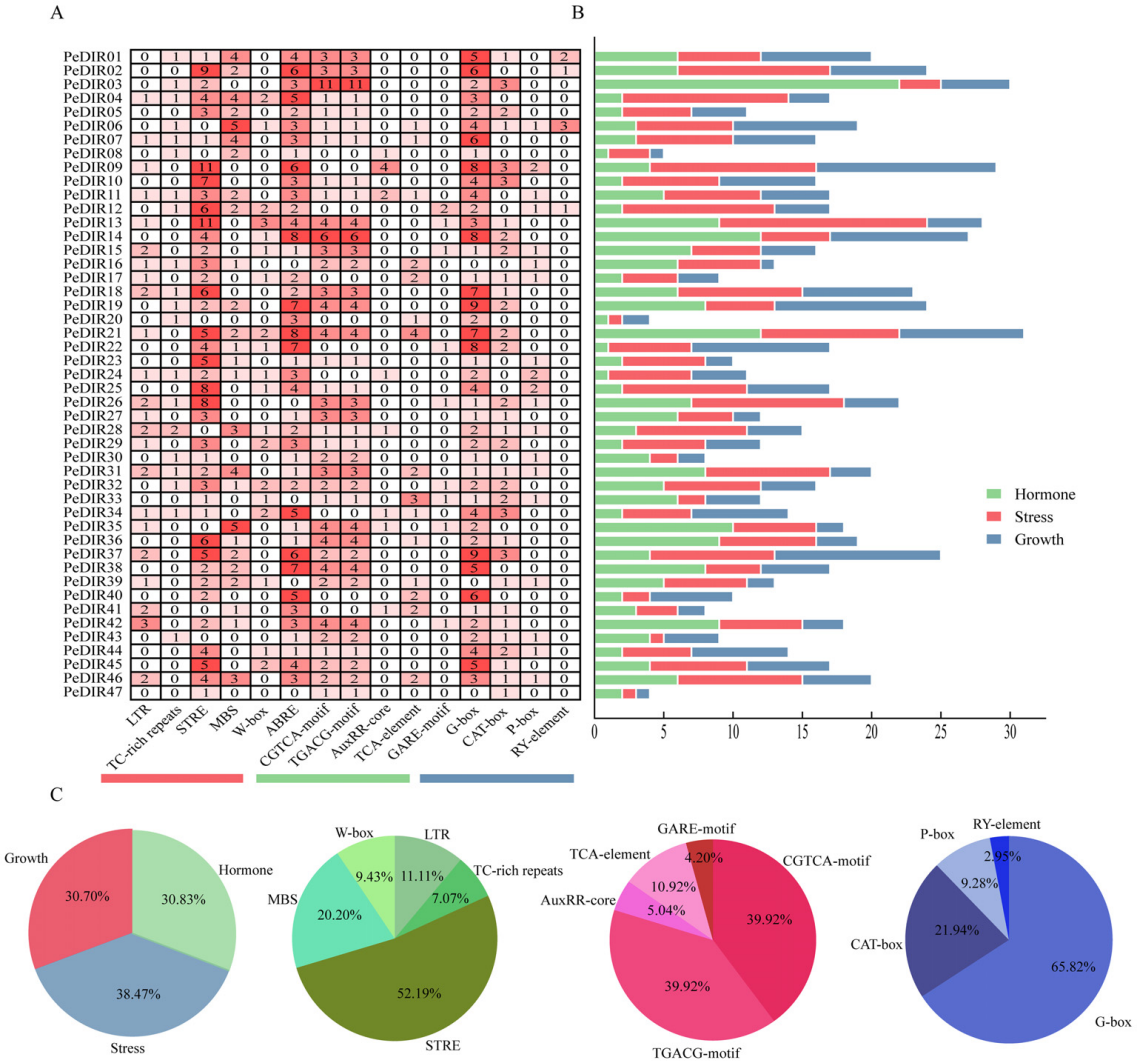
*Cis*-acting elements in the promoter regions of *PeDIR* genes. **(A)** Number of *cis*-acting elements identified in each *PeDIR* gene. **(B)** Distribution of the three primary types of *cis*-acting elements in *PeDIR* genes. **(C)** Statistical analysis of the different types of *cis*-acting elements.

The analysis of *cis*-acting elements in the 1500 bp promoter regions of *PeDIR* genes identified three primary categories: elements responsive to hormones, stress, and growth and development (**Figure 4A**). Hormone-responsive elements accounted for 41.2% and predominantly included ABRE (ABA response), TGACG and CGTCA (MeJA response), TCA-element (SA response), and GARE (GA response). Among these, ABRE, TGACG, and CGTCA were the most abundant, representing 37%, 25%, and 25%, respectively. Additionally, *PeDIR* genes contained a considerable number of stress-responsive elements, with STRE being the most prevalent, accounting for 52.2% of all stress-responsive elements. Growth and development elements, such as G-box and CAT-box, were also identified (**Figures 4B, C**). These findings suggest that *PeDIR* genes may play a significant role in the rapid growth of Moso bamboo, as well as in hormone responses and abiotic stress regulation.

### 3.5 Transcriptome expression profile of *PeDIR* genes in response to hormonal and abiotic stresses

**Figure 5:**
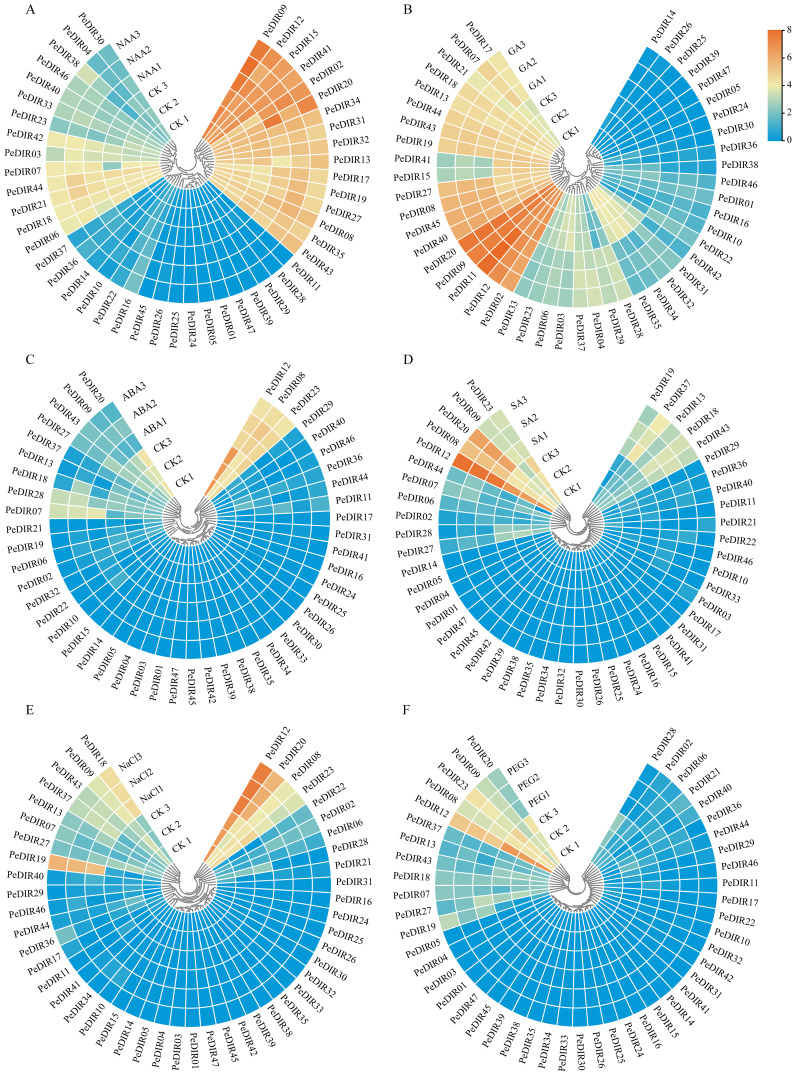
Heatmap of *PeDIR* genes expression levels under different stress treatments. **(A–D)** represent treatments with NAA, GA₃, ABA, and SA, respectively. **(E, F)** represent high-salinity and drought treatments, respectively.

To further explore the expression profiles of *PeDIR* genes under hormonal influence, transcriptome data from four different treatments (ABA, GA_3_, NAA, and SA) were analyzed. The results revealed distinct expression patterns among *PeDIR* genes in response to various hormone treatments. For instance, under ABA treatment, *PeDIR07*, *PeDIR08*, and *PeDIR28* displayed upregulation, whereas *PeDIR09*, *PeDIR12*, *PeDIR20*, *PeDIR27*, and *PeDIR37* were downregulated (**Figure 5A**). In contrast, under GA_3_ and SA treatments, *PeDIR08*, *PeDIR09*, *PeDIR12*, *PeDIR20*, and *PeDIR37* were significantly upregulated (**Figures 5B, C**). Additionally, during NAA treatment, *PeDIR02*, *PeDIR09*, and *PeDIR12* showed increased expression levels (**Figure 5D**).

Based on available transcriptome data, the expression of *PeDIR* genes during drought and salinity stresses was also examined. The results revealed significant expression changes in certain genes in response to these stresses. Overall, most genes exhibited relatively stable expression levels before and after treatments. However, under salinity stress, *PeDIR09*, *PeDIR12*, *PeDIR20*, and *PeDIR43* were notably upregulated. In contrast, under drought stress, *PeDIR12*, *PeDIR20*, and *PeDIR43* were significantly downregulated (**Figures 5E, F**). These findings suggest that these genes likely play specific regulatory roles in Moso bamboo’s responses to salinity and drought stresses.

### 3.6 Short time-series expression miner of *PeDIR* genes

**Figure 6:**
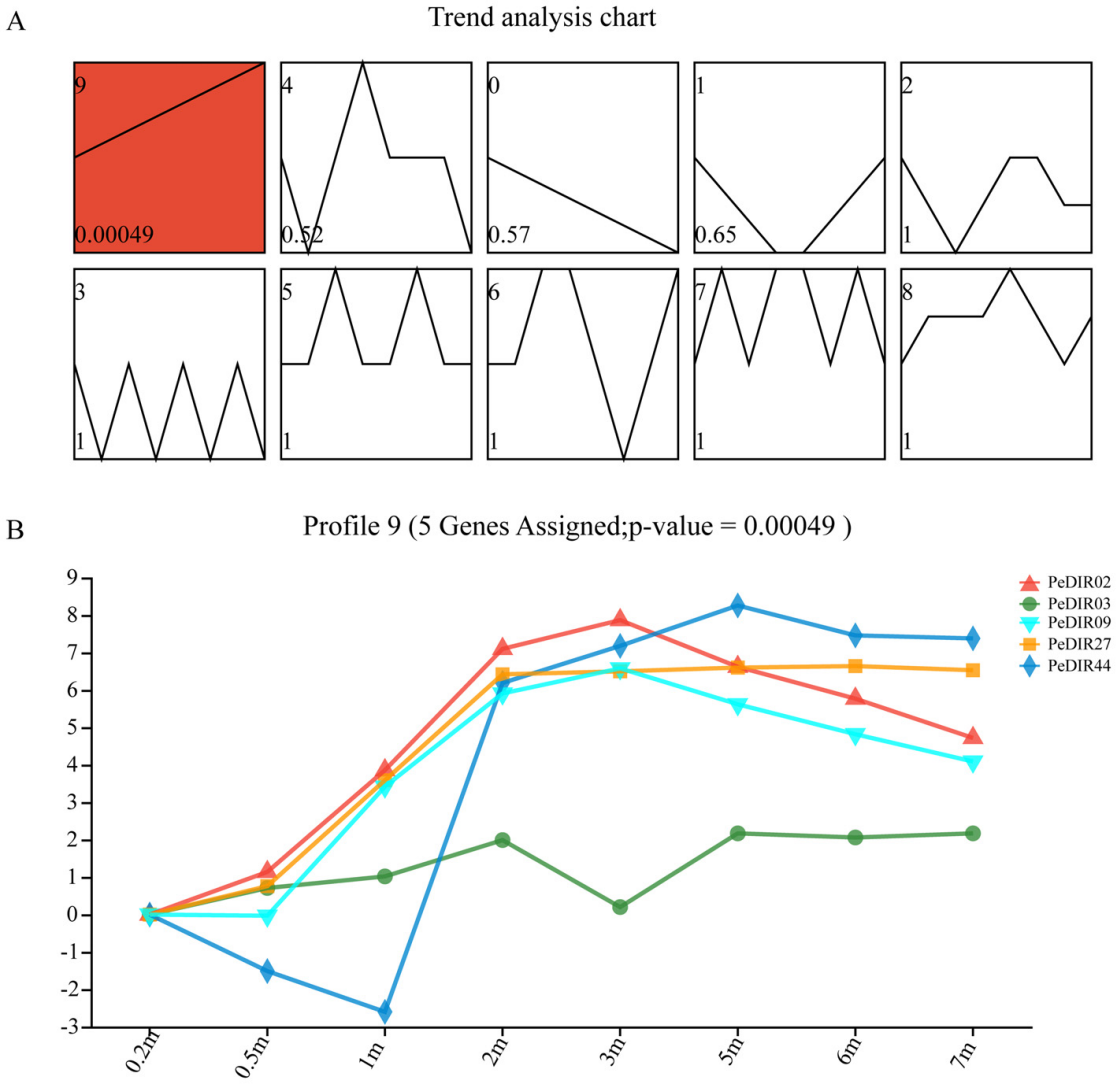
STEM analysis of *PeDIR* genes at different growth heights. **(A)** Red boxes indicate significantly expressed genes. All 10 profiles are shown in the top-left corner, with P-values displayed in the bottom-left corner. **(B)** Expression levels of significantly expressed *PeDIR* genes in Profile 9.

The expression profiles of *PeDIR* genes in Moso bamboo shoots at varying heights (0.2–7 m) were also analyzed using STEM software. The analysis identified five *PeDIR* genes (*PeDIR02*, *PeDIR03*, *PeDIR09*, *PeDIR27*, and *PeDIR44*), highlighted in red in profile 3, as significantly expressed (**Figure 6A**). These genes exhibited a marked upregulation of expression at heights between 0.2 and 2 m, followed by relatively stable expression levels from 2 to 7 m (**Figure 6B**). These findings suggest that these genes are closely associated with the regulation of rapid growth in Moso bamboo. They likely contribute to critical processes such as lignin biosynthesis and cell wall structure modulation during early shoot development.

### 3.7 qRT-PCR analysis of *PeDIR* genes

**Figure 7:**
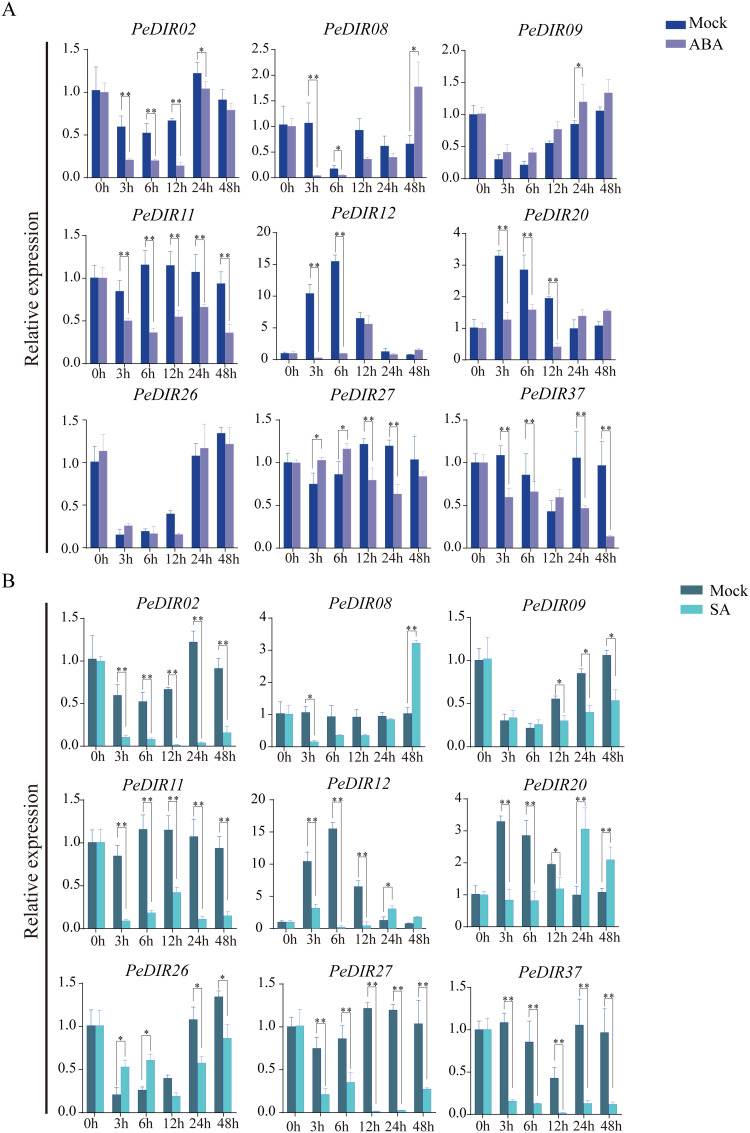
Expression pattern analysis of *PeDIR* genes under ABA **(A)** and SA **(B)** treatments. Asterisks indicate significant differences compared to the control (*P ≤ 0.05; **P ≤ 0.01).

To further validate the expression profiles of *PeDIR* genes under different hormonal conditions, the expression responses of nine *PeDIR* genes were analyzed by qRT-PCR following ABA and SA treatments. During ABA treatment, *PeDIR02*, *PeDIR11*, *PeDIR12*, and *PeDIR37* were consistently downregulated across all time points, while *PeDIR08* and *PeDIR20* initially exhibited downregulation followed by upregulation. *PeDIR26* and *PeDIR37* displayed fluctuating expression patterns (**Figure 7A**). Under SA treatment, most *PeDIR* genes were downregulated at all time points, except for *PeDIR08* and *PeDIR20*, which were downregulated during the early stages but significantly upregulated between 24 and 48 hours (**Figure 7B**).

**Figure 8:**
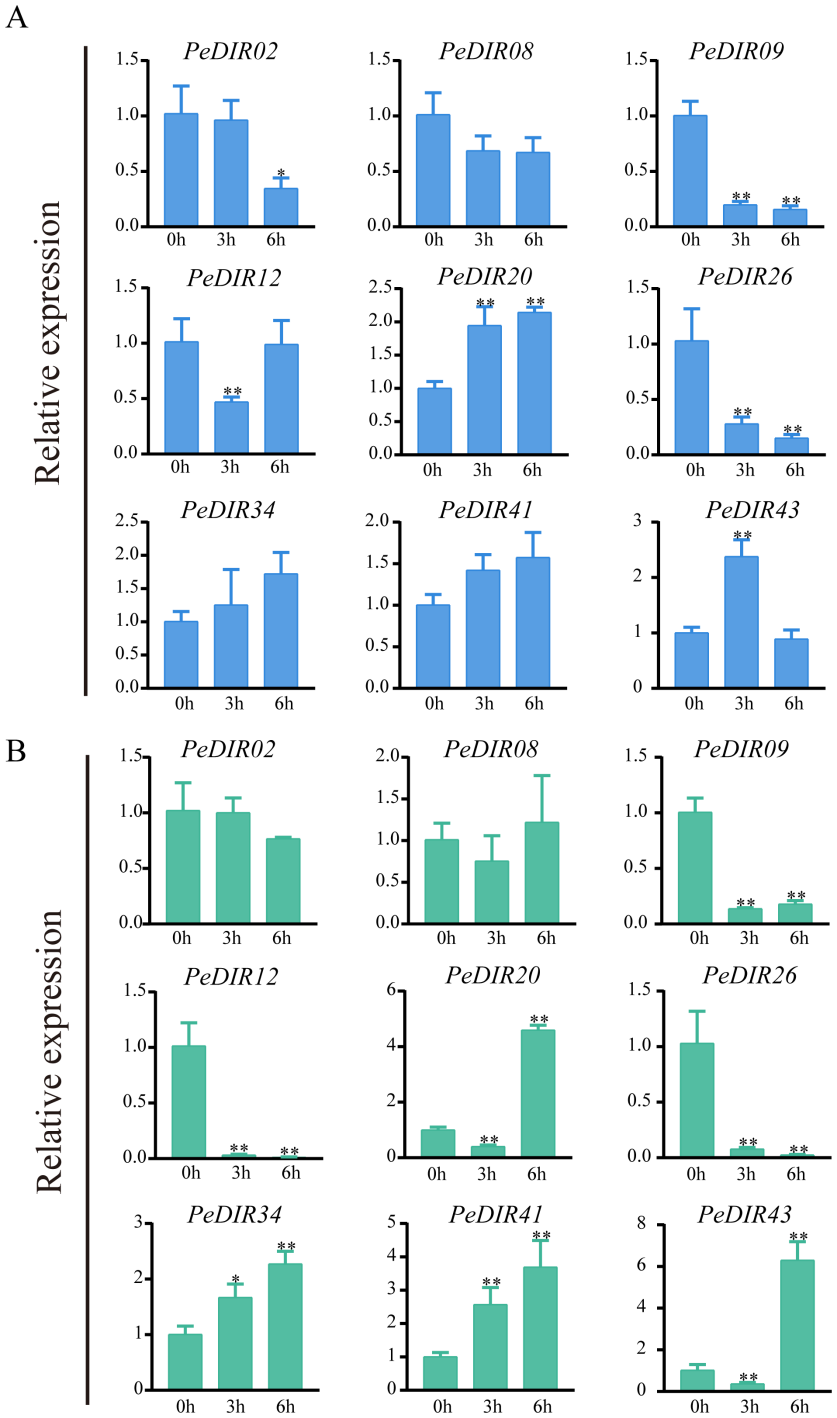
Expression pattern analysis of *PeDIR* genes under drought **(A)** and high-salinity **(B)** treatments. Asterisks indicate significant differences compared to the control (*P ≤ 0.05; **P ≤ 0.01).

Under drought and high-salinity stresses, qRT-PCR analysis confirmed the expression profiles of *PeDIR* genes, which strongly correlated with the transcriptome data. Specifically, *PeDIR08* and *PeDIR09* exhibited significantly reduced expression levels after 6 hours of treatment. In contrast, *PeDIR20* and *PeDIR43* were notably upregulated under both drought and high-salinity stresses, with *PeDIR20* showing a marked increase, particularly 6 hours after salinity stress treatment (**Figure 8A**). Additionally, *PeDIR12* and *PeDIR26* were consistently downregulated under both stress conditions, whereas *PeDIR34* and *PeDIR41* were upregulated specifically under high-salinity stress (**Figure 8B**). These findings highlight the diverse expression profiles of *PeDIR* genes under drought and salinity stresses, underscoring the complex regulatory roles of this gene family in Moso bamboo’s abiotic stress responses.

### 3.8 Secondary and tertiary structure prediction of PeDIR proteins

**Figure 9:**
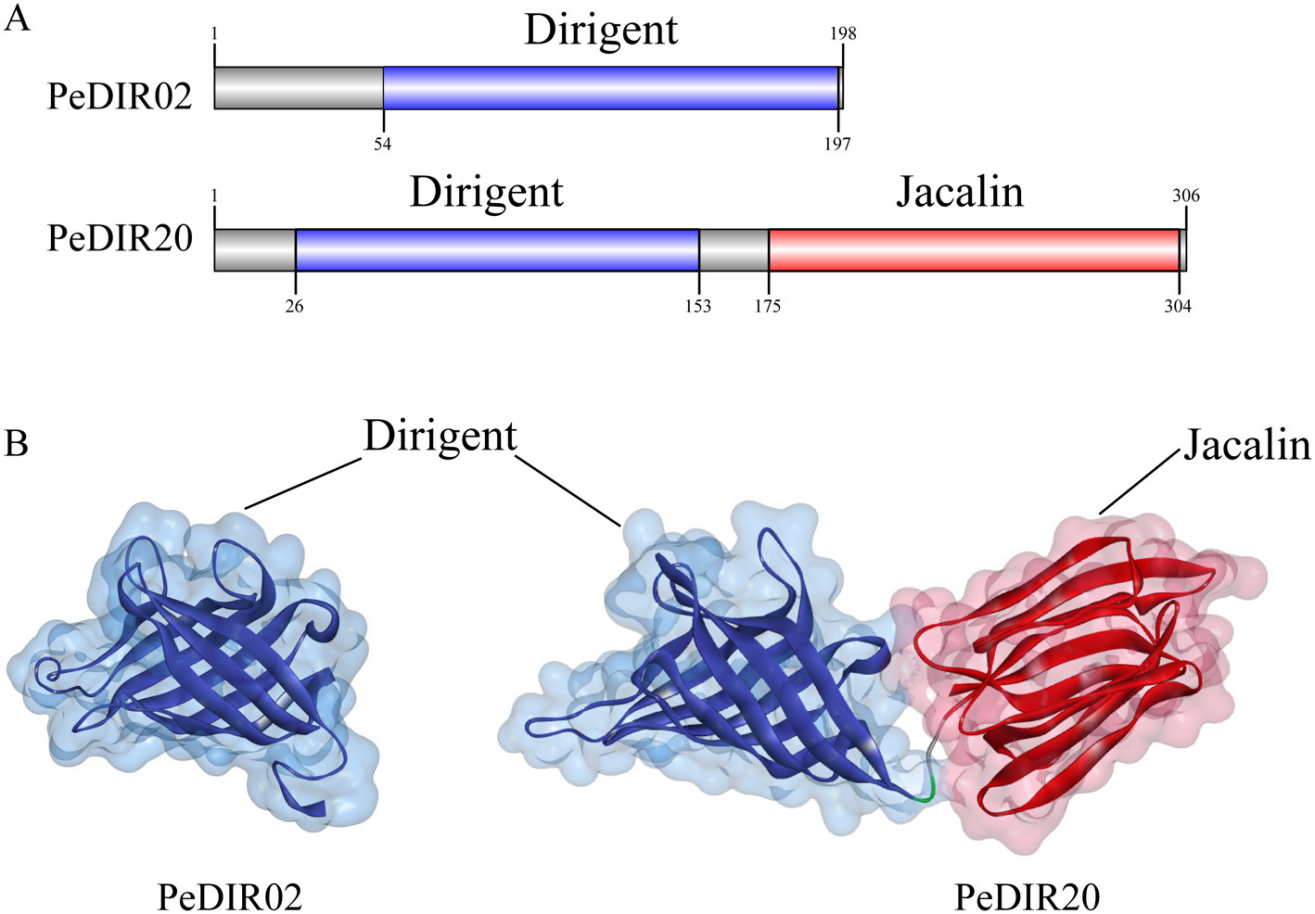
Secondary and tertiary structures of PeDIR02 and PeDIR20 proteins. **(A)** Conserved domains of PeDIR02 and PeDIR20. **(B)** Three-dimensional structures of PeDIR02 and PeDIR20.

To better understand the structural basis underlying the functional diversity of PeDIR proteins, domain prediction analysis was performed. This analysis revealed that all PeDIR proteins contain a Dirigent domain. Additionally, PeDIR12, PeDIR20, and PeDIR43 possess an additional Jacalin domain. Based on these domain differences, the PeDIR protein family can be categorized into two types: Type I, which contains only the Dirigent domain (e.g., PeDIR02), and Type II, which contains both Dirigent and Jacalin domains (e.g., PeDIR20) (**Figure 9A**). To further investigate the spatial characteristics of these domains, representative members from each type were selected for tertiary structure prediction and modeling (**Figure 9B**). The results revealed structural similarities between the Dirigent and Jacalin domains, both of which are composed of multiple β-strands. Specifically, the Dirigent domain consists of eight antiparallel β-strands arranged in a barrel-like structure. Notably, PeDIR20, which possesses both Dirigent and Jacalin domains, is hypothesized to exhibit functional properties characteristic of both the DIR and JRL families.

### 3.9 Subcellular localization and homodimer detection of PeDIR proteins

**Figure 10:**
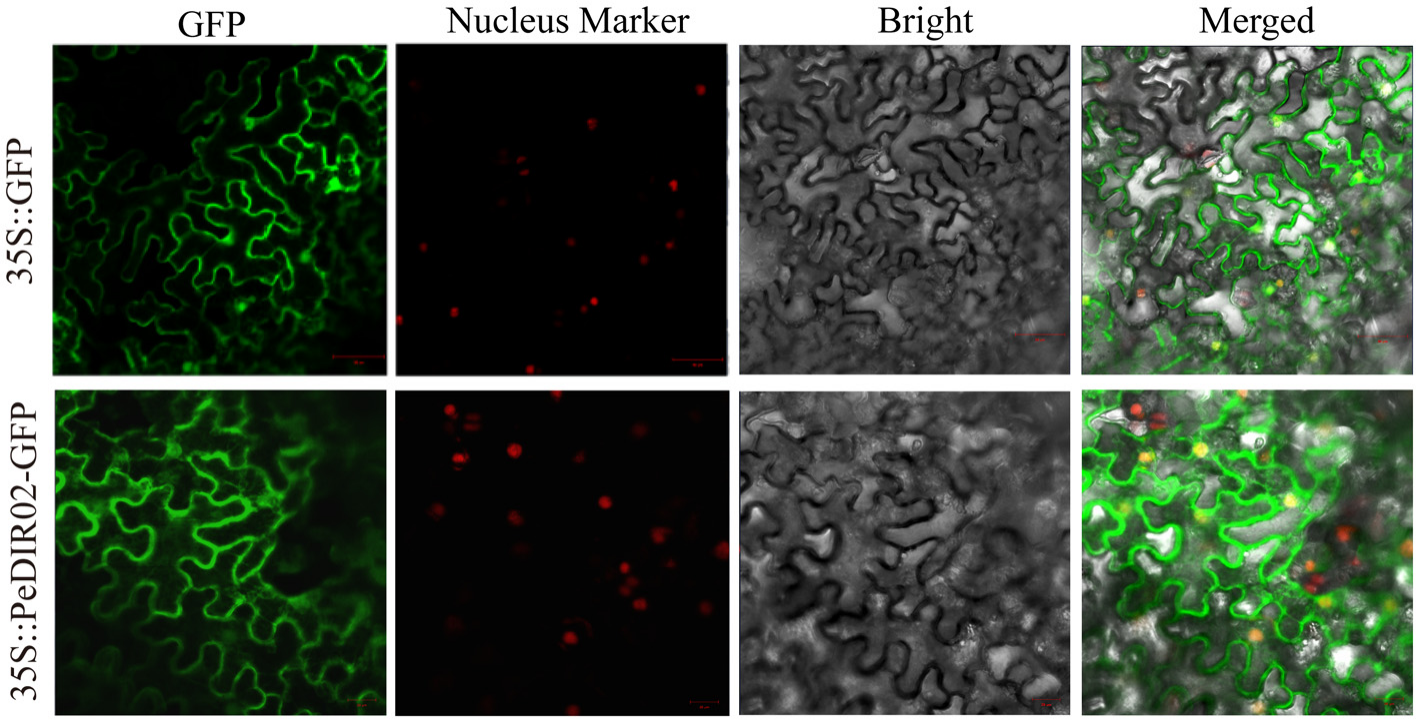
Subcellular localization of PeDIR02 in tobacco leaves. GFP, green fluorescent protein.

**Figure 11:**
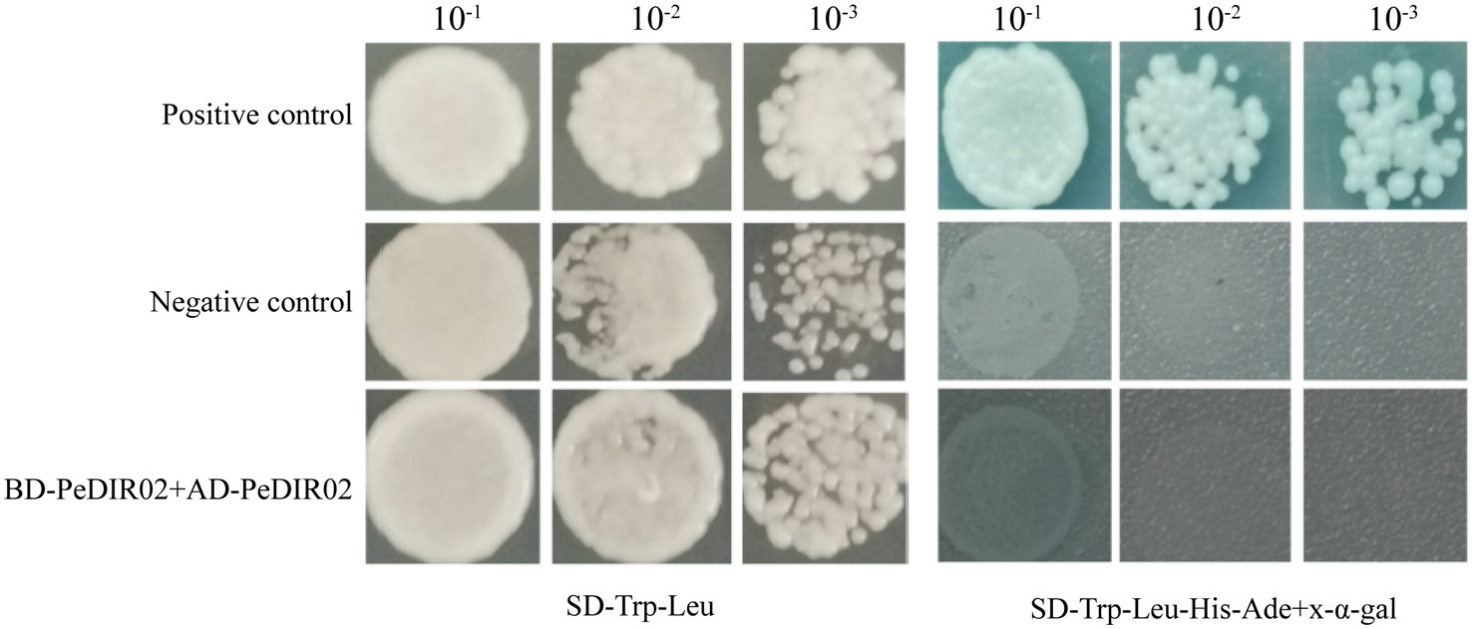
Confirmation of homodimer structures of PeDIR02 in yeast two-hybrid (Y2H) assays.

To examine the subcellular localization of PeDIR02 proteins, the coding sequence (CDS) of PeDIR02 was fused with GFP, and its expression in tobacco epidermal cells was transiently driven by the CaMV35S promoter. GFP alone was used as a control. The results showed that PeDIR02 was primarily localized to the cell membrane, with minor distributions in the cytoplasm and nucleus (**Figure 10**). To test the self-interaction of PeDIR02 proteins, the genes were cloned into appropriate vectors and introduced into a yeast strain. Growth on SD/-Trp/-Leu medium confirmed successful transformation. However, on SD/-Trp/-Leu/-His/-Ade + X-α-Gal medium, PeDIR02 did not exhibit normal growth or a blue color reaction, indicating the absence of self-interaction (**Figure 11**).

### 3.10 Regulatory network construction of *PeDIR* genes

**Figure 12:**
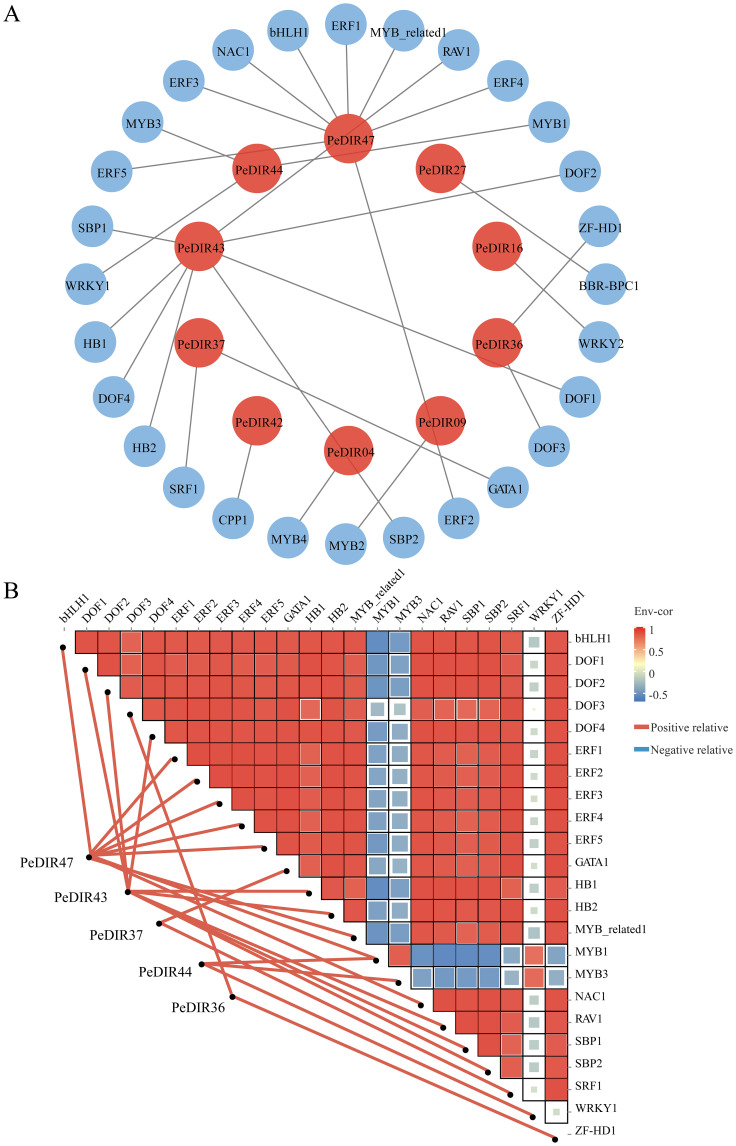
Regulatory network of upstream transcription factors for *PeDIR genes*. **(A)** Predicted regulatory network of upstream transcription factors for *PeDIR genes*. **(B)** Correlation heatmap between *PeDIR genes* and transcription factors.

To investigate the transcriptional regulation of *PeDIR* genes, a putative regulatory network involving transcription factors (TFs) was constructed. Twenty-eight TFs, primarily from the DOF, MYB, ERF, and WRKY families, were identified as regulators of 10 *PeDIR* genes. Among these, *PeDIR43*, *PeDIR44*, and *PeDIR47* showed strong associations with multiple TFs, underscoring their central roles within the network (**Figure 12A**; **Supplementary Table S4**). Analysis of dynamic interactions between key TFs and *PeDIR* genes revealed that all TFs act as positive regulators of their target genes. Notably, aside from MYB1 and MYB3, significant positive correlations were observed among the TFs, indicating coordinated regulation of *PeDIR* gene expression (**Figure 12B**). These results suggest that the PeDIR gene family is collectively regulated by multiple TFs, contributing to Moso bamboo’s adaptation to environmental stresses and its growth processes.

## 4 Discussion

The DIR gene family is widely involved in the biosynthesis of lignin and lignans and is almost ubiquitously present in vascular plants. This family has been identified in various monocots, including rice (49 members) (Liao et al., 2017), *Setaria italica* (38 members) (Gong et al., 2023), and *Schisandra chinensis* (34 members) (Dong et al., 2021). In this study, we identified a total of 47 *PeDIR* members in Moso bamboo. This number is comparable to other Poaceae species but significantly higher than in dicots such as *Arabidopsis thaliana* (25 members) (Paniagua et al., 2017), *Solanum melongena* (24 members) (Zhang et al., 2022a), and *Isatis indigotica* (19 members) (Li et al., 2014). These differences suggest that the DIR gene family may have undergone lineage-specific expansion in monocots.

Structurally, most *PeDIR* genes contain 2–5 introns, whereas DIR genes in dicots like *Arabidopsis* and poplar (*Populus trichocarpa*) have simpler structures, often lacking introns (Paniagua et al., 2017; Li et al., 2021). Furthermore, *PeDIR* and *OsDIR* genes are arranged in clusters within their genomes, a feature not observed in *Arabidopsis*. These structural differences support the hypothesis that the DIR gene family underwent distinct expansion mechanisms following the divergence of monocots and dicots. Gene duplication events, including tandem duplications, segmental duplications, and whole-genome duplications, have facilitated the formation of new genes and regulatory pathways, enabling plants to adapt to diverse environments (Freeling, 2009; Xuan et al., 2024).

In this study, 67.6% of *PeDIR* genes were located in tandemly duplicated regions, similar to the 72.6% observed in rice (Paniagua et al., 2017; Li et al., 2021), indicating that tandem duplication may have played a significant role in the expansion of *DIR* genes in monocots. Synteny analysis with other Poaceae species revealed extensive collinearity between Moso bamboo and rice or maize, but fewer syntenic relationships with *B. distachyon*. This suggests that *PeDIR* genes in Moso bamboo share closer phylogenetic relationships with those in rice and maize than with *B. distachyon*. These findings are consistent with previous studies on *PeHAK* and *PeCPP* genes (Guo et al., 2024; Tan et al., 2024).

Phylogenetic analysis classified the *PeDIR* genes into three subfamilies: DIR-b/d, DIR-a, and DIR-e. Motif and gene structure analyses further supported this classification, indicating potential functional differentiation among the subfamilies. Previous studies have shown that *DIR-a* genes play critical roles in the stereoselective coupling of lignin and lignan intermediates, thereby contributing to lignin biosynthesis (Gasper et al., 2016; Corbin et al., 2018).

To explore the structural and functional characteristics of *PeDIR* genes, three-dimensional models of two representative DIR proteins, PeDIR02 and PeDIR20, were constructed. PeDIR02, a member of the DIR-b/d subfamily, forms a single barrel-like structure composed of eight antiparallel β-strands. In contrast, PeDIR20, classified under the DIR-a subfamily, contains both a Dirigent domain and a Jacalin domain, forming two distinct barrel-like structures. Previous studies have shown that DIR proteins often function by forming homodimers or trimers (Halls et al., 2004; Kim et al., 2012). Yeast two-hybrid assays revealed that PeDIR02 cannot self-interact to form dimers, indicating that it does not function as a homodimer. Subcellular localization analysis showed that PeDIR proteins are primarily located on the cell membrane, with minor distributions in chloroplasts and the cytoplasm. Experimental validation confirmed that PeDIR02 is predominantly localized on the cell membrane. This result aligns with the subcellular localization of SiDIR proteins in foxtail millet (Gong et al., 2023), suggesting that PeDIR proteins may primarily function at the cell membrane. These insights provide valuable information on the functional mechanisms of DIR proteins in Moso bamboo, laying a foundation for further studies.

*DIR* genes play crucial roles in growth, development, and environmental adaptation, particularly in hormone and abiotic stress responses (Cai-Qiu et al., 2010; Peng et al., 2013). While *DIR* gene expression patterns vary across species, numerous studies have reported their regulation under hormonal treatments and abiotic stresses in plants such as potato (Jia et al., 2023), tomato (FengQiong et al., 2022), and barley (Luo et al., 2022). This study found that 22 *PeDIR* genes responded to at least two hormones, with two genes consistently upregulated under SA treatment and four genes significantly upregulated under ABA treatment. These findings align with those in rice, where hormone-responsive *DIR* genes are similarly regulated (Liao et al., 2017). Notably, *PeDIR08* and *PeDIR20* were significantly upregulated in the later stages of ABA and SA treatments, suggesting that these genes play key roles in hormonal response pathways in Moso bamboo.

Under abiotic stress conditions, *PeDIR* genes exhibited diverse expression patterns in response to drought and salinity stresses. Most *PeDIR* genes were upregulated under these stresses, with *PeDIR20* and *PeDIR43* showing particularly significant upregulation. Interestingly, both *PeDIR20* and *PeDIR43* belong to the DIR-a subfamily, which is closely associated with lignin and lignan biosynthesis. This association likely enhances protective functions under stress conditions (Cheng et al., 2018; Li et al., 2022). These findings suggest that DIR-a subfamily members, especially *PeDIR20* and *PeDIR43*, may play critical roles in enhancing stress tolerance in Moso bamboo, contributing to its growth stability under adverse conditions.

To investigate the regulatory mechanisms of the PeDIR gene family, this study constructed and analyzed a transcriptional regulatory network for *PeDIR* genes. *Cis*-regulatory element analysis identified numerous elements associated with hormone responses (e.g., ABRE, TGACG, CGTCA, and TCA-element), abiotic stresses (e.g., STRE and MBS), and growth and development (e.g., G-box and P-box) (Kumari et al., 2025). Predictions of the transcriptional regulatory network indicated that transcription factors such as ERF, DOF, and MYB play key roles in regulating *PeDIR* gene expression, with evidence of positive synergistic interactions. ERF and MYB transcription factors have been shown to play critical roles in abiotic stress responses, such as drought, salinity, and cold (Xiaoxiao et al., 2022; Ziming et al., 2024). Similarly, the DOF family has been reported to positively regulate hormone signaling and abiotic stress responses in plants such as wheat (Yue et al., 2020). In Moso bamboo, these positive regulatory transcription factors likely interact with *cis*-elements in the promoters of *PeDIR* genes, coordinating their expression under hormone signaling and abiotic stress conditions to enhance environmental adaptability and regulate growth and development.

This study significantly advances our understanding of the DIR gene family in Moso bamboo, particularly highlighting the roles of specific genes such as *PeDIR08* and *PeDIR20* in rapid shoot growth and stress responses. The findings provide a valuable foundation for developing bamboo varieties capable of thriving in challenging environmental conditions, such as drought and high salinity. By enhancing the expression of these genes, it may be possible to improve bamboo’s growth rate and stress resistance, leading to more sustainable and efficient cultivation practices. Additionally, these insights offer a comparative framework for understanding the functions of *DIR* genes in other plant species and inform strategies for improving stress tolerance in crops.

Future research on the PeDIR gene family in Moso bamboo should focus on quantifying lignin to elucidate its role in cell wall development, analyzing hormone interactions to uncover regulatory mechanisms, and conducting pathway analyses to explore the complex signaling networks involving *DIR* genes, hormones, and stress responses. These studies will provide valuable insights into the functional roles of *DIR* genes and help enhance bamboo’s resilience and productivity.

## 5 Conclusion

This study comprehensively identified and characterized the PeDIR gene family in Moso bamboo, identifying a total of 47 members. Detailed analyses were performed on their gene structures, phylogenetic relationships, promoter *cis*-acting elements, and transcription factor regulatory networks. *PeDIR* genes demonstrated significant roles in rapid shoot growth, hormonal responses (e.g., ABA and SA), and abiotic stress responses (e.g., drought and salinity). These findings provide a crucial foundation for understanding the functions of *PeDIR* genes in growth and stress adaptation. Future research could leverage gene editing and biotechnological approaches to optimize the expression of key *PeDIR* genes, paving the way for the development of bamboo varieties with enhanced stress resistance and adaptability to climate change.

## Data Availability

The original contributions presented in the study are included in the article/**Supplementary Material**. Further inquiries can be directed to the corresponding authors.
